# Ologen Implant versus Mitomycin C for Trabeculectomy: A Systematic Review and Meta-Analysis

**DOI:** 10.1371/journal.pone.0085782

**Published:** 2014-01-20

**Authors:** Miao He, Wei Wang, Xiulan Zhang, Wenyong Huang

**Affiliations:** Zhongshan Ophthalmic Center, State Key Laboratory of Ophthalmology, Sun Yat-Sen University, Guangzhou, People’s Republic of China; Zhongshan Ophthalmic Center, China

## Abstract

**Objective:**

To evaluate the application of the Ologen implant compared to mitomycin C (MMC) on the outcome of trabeculectomy and to examine the balance of risks and benefits.

**Methods:**

A systematic literature search (Pubmed, Embase, the Cochrane Library, and the Chinese Biomedicine Database) was performed. Randomized controlled trials comparing the Ologen implant with MMC in trabeculectomy were selected. The efficacy measures were the weighted mean differences (WMDs) for the intraocular pressure reduction (IOPR), the reduction in glaucoma medications, and the relative risks (RRs) for success rates. The tolerability measures were RRs for adverse events. The pooled effects were calculated using the random-effects model.

**Results:**

Seven randomized controlled trials including 227 eyes were included in this meta-analysis. The WMDs of the IOPR comparing the Ologen group with the MMC group were −2.98 (95% Cl: −5.07 to −0.89) at one month, −1.41 (−3.72 to 0.91) at three months, −1.69 (−3.68 to 0.30) at six months, −1.94 (−3.88 to 0.01) at 12 months, and 0.65 (−2.17 to 0.47) at 24 months. There was no statistically significance except at one and 12 months after surgery. No significant difference in the reduction in glaucoma medications or complete and qualified success rates were found. The rates of adverse events also did not differ significantly between Ologen and MMC.

**Conclusions:**

The Ologen implant is comparable with MMC for trabeculectomy in IOP-lowering efficacy, reduction in the number of glaucoma medications, success rates, and tolerability. However, the results should be interpreted cautiously since relevant evidence is still limited, although it is accumulating. Further large-scale, well-designed randomized controlled trials are urgently needed.

## Introduction

Since it was introduced in 1968 by Cairns, trabeculectomy remains the most common surgical procedure for the treatment of glaucoma [1], [2], [3]. The method was developed further over subsequent decades to address various problems. Wound healing and scar formation causing fibrosis and the obstruction of aqueous outflow remain the most common reason for the failure of trabeculectomy [4], [5]. To resolve this problem, several agents have been used. In 1990, MMC was applied as an anti-metabolite during trabeculectomy. MMC is an antitumor antibiotic isolated from *Streptomycin caepitorus*. It inhibits the synthesis of DNA, cellular RNA, and protein by inhibiting the synthesis of collagen by fibroblasts [6], [7].

MMC was originally used as a systemic chemotherapeutic agent; it has been widely used in ophthalmic practice both intraoperatively and postoperatively for enhancing the success rate of glaucoma filtration surgery. Recent systematic reviews have demonstrated significant enhancement of success rates and postoperative IOP through the intraoperative use of MMC during glaucoma filtering surgery [6], [8], [9]. However, it is frequently accompanied by short- and long-term complications such as hypotony, bleb leaks, cataract formation, avascular filtering blebs, thinning of the conjunctiva, subsequent blebitis, and endophthalmitis [10]. Therefore, there is still an urgent need for a safer alternative for fibrosis control.

The Ologen implant was developed aiming at replacing MMC for trabeculectomy. It is a disc-shaped porcine-derived biodegradable collagen matrix that has been developed to prevent excessive scarring after trabeculectomy [2], [11]. When inserted under the conjunctiva at the time of trabeculectomy, it not only acts as a reservoir but also helps to separate mechanically the conjunctiva and episcleral surface and prevent adhesions between them [12], [13]. Recently, many comparative controlled trials have compared the efficacy and tolerability of trabeculectomy augmented with Ologen versus trabeculectomy plus MMC, but the results are not completely consistent. Some studies [14], [15], [16], [17], [18], [19] have found that the techniques were comparable in lowering IOP efficacy, whereas others have suggested that Ologen is inferior to MMC [20], [21], [22].

These conflicting results have made it difficult to draw conclusions that could be applied in clinical practice. Therefore, the aim of this study was to undertake a systematic review and meta-analysis to evaluate the application of the Ologen implant compared to mitomycin C (MMC) on the outcome of trabeculectomy and to examine the balance of risks and benefits.

## Materials and Methods

This systematic review and meta-analysis were performed according to a predetermined protocol described in the next paragraph, and the standard systematic review guidelines, as outlined by the Cochrane Reviewers’ Handbook and the PRISMA (Preferred Reporting Items for Systematic Review and Meta-Analyses) statement (Table S1), were followed in all stages of the process [23], [24].

### 1. Search Strategy

Reports of clinical trials comparing trabeculectomy with Ologen and with MMC were identified through a systematic search consisting of (i) an electronic search of Pubmed, Embase, the Cochrane Library, and the Chinese Biomedicine Database; (ii) manual searches of the reference lists of original reports and review articles retrieved through the electronic searches; and (iii) extensive Internet searches, including manufacturers’ databases, websites of professional associations, and the Google Scholar search engine. Searches were conducted using the keywords “trabeculectomy,” “sclerectomy,” “Ologen implant,” “OculusGen,” “iGen,” “collagen matrix implant,” “MMC,” and “mitomycin C.” No language or date restrictions were applied. The computerized searches covered the period from 1966 to July 2013. The retrieved studies were imported into Refworks (version 1.0; Refworks, Bethesda, MD), where duplicate articles were manually deleted. The titles and abstracts of the remaining studies were independently scanned by two reviewers (W.W. and M.H.). The full texts of the potentially relevant reports were then read to determine whether they met the inclusion criteria.

### 2. Inclusion and Exclusion Criteria

Published and unpublished trials fulfilling the following selection criteria were included in the present meta-analysis: (1) study design–randomized controlled clinical trials (RCTs); (2) population–adult patients (>18 years) with uncontrolled glaucoma undergoing trabeculectomy; (3) intervention–Ologen was compared with intraoperative MMC of any concentration and dose; (4) outcome variables–at least one of the following outcome variables: IOPR, reduction in glaucoma medications, complete and qualified success rates, or incidence of adverse events; and (5) and a follow-up time of at least six months. The following were excluded: (1) studies that involved other types of glaucoma surgery, such as non-penetrating glaucoma surgery and (2) studies that included pediatric cases or patients with repeated glaucoma surgery. Where multiple publications based on the same group of patients were identified, the report with the largest number of patients was used.

### 3. Data Extraction

Data were extracted from each RCT by two independent reviewers (W.W. and M.H.). Any discrepancies between the two independent data extractions were resolved by discussion to reach a consensus among all authors. For the eligible studies, the following data were extracted: (1) general characteristics (title, first author, journal title, and year of publication); (2) methodology (type of study, country of origin, sequence generation, allocation concealment, masking or blinding, incomplete outcome data, selective reporting, and other sources of bias); (3) subjects (recruitment site, enrollment periods, inclusion criteria, exclusion criteria, and general patient characteristics); (4) interventions (concentration of MMC and exposure time); (5) outcomes (measurement, follow-up time and loss of follow-up); (6) analysis (statistical methods); and (7) results (quantitative results and qualitative results). If the appropriate data were not obtainable, we requested the data from the study's investigators.

### 4. Quality Assessment

The methodological quality of each study was assessed using the risk-of-bias tool outlined in the Cochrane Handbook for Systematic Reviews of Interventions (version 5.1.0) [23]. Two reviewers (W.W. and M.H.) subjectively reviewed all studies and assigned a value of “high,” “low,” or “unclear” to the following: (a) selection bias; (b) blinding; (c) attrition bias; (d) reporting bias; and (e) other biases.

### 5. Outcome Measures

The primary outcome was the IOP (IOPR) reduction from preoperative to postoperative. When authors reported the mean and SD of IOP and IOPR, we used them directly. When not available, we computed them according to the methods described in the Cochrane Handbook for Systematic Reviews of Interventions: IOPR = IOP_baseline_-IOP_endpoint_ and SD_IOPR_ = (SD^2^_baseline_+SD^2^_endpoint_–SD_baseline_×SD_endpoint_)^1/2^ [23], [25], [26]. When the difference in means (MD) and its *t*-value [*t*-value also can be obtained from a computer by entering = tinv (*P* value, N_treat_+N_control_−2) into any cell in a Microsoft Excel spreadsheet] were reported, SD = MD−*t* ^−1^×(N^−1^_treat_+N^−1^_control_)^−1/2^ [23]. The secondary outcome measure that we have reviewed was the difference in the reduction in glaucoma medications. For efficacy, the proportion of complete success and qualified success was also used. Complete success was defined as target endpoint IOP (usually <21 mm Hg) without medications, and qualified success was defined as target endpoint IOP with or without medications. The fourth outcome was adverse event rates in either group, including wound leaks, hyphema, a shallow anterior chamber, hypotony, choroidal effusion, an encapsulated bleb, blebitis, hypotony maculopathy, and implant exposure.

### 6. Statistical Analysis

Not all of the trials reported on all the outcomes of interest. For each comparison and outcome, we undertook separate meta-analyses. Outcome measures were assessed on an intent-to-treat basis. Considering the different clinical characteristics among study groups and the variation of sample sizes, we assumed that heterogeneity was present even when no statistical significance was identified, and we decided to combine data by using a random-effects model to achieve more conservative estimates [27].

Statistical heterogeneity was analyzed using a chi-square test. The *I^2^* statistic was calculated to assess heterogeneity between studies (P<0.10 was considered representative of significant statistical heterogeneity) [28].

Dichotomous data were presented as the relative ratio (RR) with a 95% confidence interval (CI). Weighted mean differences (WMD) with a 95% CI were calculated for continuous variables. Both RRs and WMDs were considered statistically significant at the P<0.05 level. One-way sensitivity analyses were performed by iteratively removing one study at a time to assess the stability of the meta-analysis results. Only outcomes of interest that were reported in >5 studies were included in the sensitivity analysis. Potential publication bias was estimated by both visually evaluating a funnel plot and the Egger test [29]. The analyses were conducted using the Stata software package (version 11.0; Stata Corp., College Station, TX).

## Results

### 1. Study Selection

**Figure 1:**
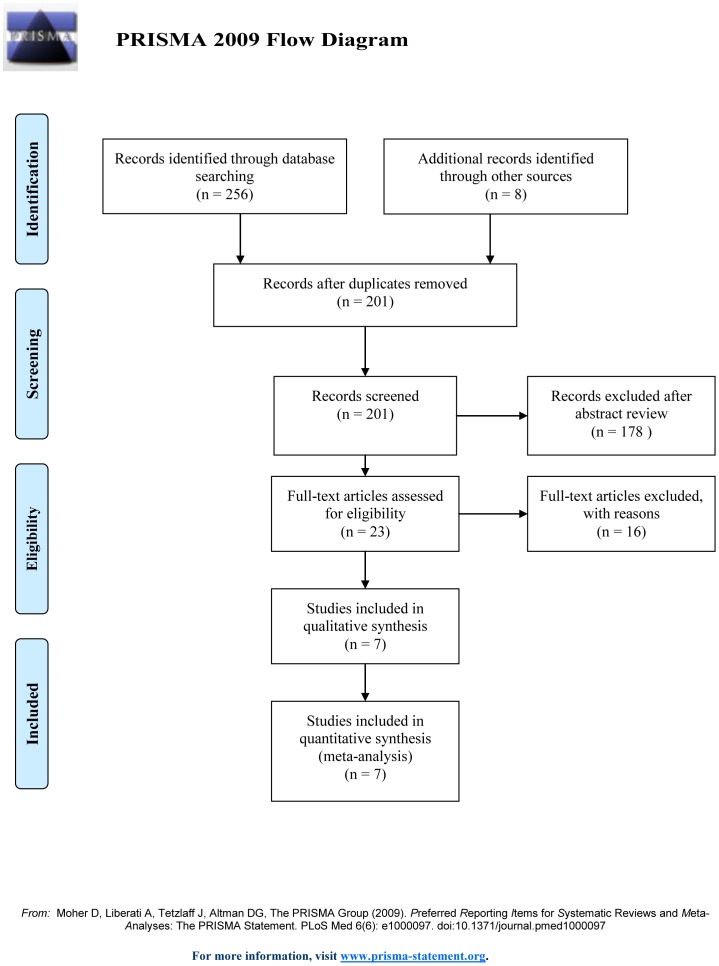
Flow of randomized controlled trials included in the meta-analysis.

The selection of studies is summarized in Figure 1. A total of 264 articles were initially identified; 256 records were identified in the database search, and eight records were found in article reference lists. However, only 23 of these studies investigated the effect of Ologen on glaucoma surgery in adults. Of these 23 articles, five were non-comparative case series, nine were not trabeculectomies, one was from the same patient group, and one compared the outcomes of trabeculectomy with or without an Ologen implant. This left seven RCTs that met our inclusion criteria to be included in the final meta-analysis [14], [15], [16], [17], [18], [19], [22].

### 2. Characteristics of Eligible Studies

The RCTs were published between 2010 and 2013 and involved a total of 227 eyes (134 in the Ologen group and 143 in the MMC group). The characteristics of the eligible studies are summarized in Table 1. Two studies were done in India and one each in Egypt, the UK, Italy, Iran, and Germany. The mean age of the patients ranged from 48.0 to 65.8 years, and the percentage of male patients ranged from 40.0% to 59.4%. Sample sizes in these studies ranged from 14 to 64. The mean follow-up period ranged from six to 24 months. The dose of MMC used ranged between 0.2 and 0.4 mg/mL, and the exposure time ranged from two to three minutes. Two of the seven studies included both open- and closed-angle glaucomas, four studies included open-angle glaucoma, and one study did not provide details regarding glaucoma diagnosis.

**Table 1:** Characteristics of Randomized Controlled Trials Comparing Trabeculectomy With Ologen Implant and With MMC.

Trial (year)	Center	Location	Goup	NO. ofeyes	Endpointlength(m)	Age(year)	Sex(M/F)	Type of glaucoma				MMC	
								POAG	PACG	PXFG	Others	Con(mg/ml)	Time(min)
Senthil (2013)	single	India	Ologen	19	24	48±10	9/10	8	11	0	0		
			MMC	20	24	45±12	11/9	12	8	0	0	0.4	2
Marey (2013)	single	Egypt	Ologen	30	12	50.2±10.2	18/12	18	4	2	6		
			MMC	30	12	49.07±5.8	17/13	13	5	4	8	0.2	2
Mitra (2012)	single	UK	Ologen	28	6	61.22±12.24	16/12	19	0	6	3		
			MMC	36	6	62.43±14.43	22/14	21	0	12	3	na	na
Maheshwari (2012)	single	India	Ologen	20	12	na	na	20	0	0	0		
			MMC	20	12			20	0	0	0	na	na
Cillino (2011)	single	Italy	Ologen	20	24	65.8±6.4	12/8	13	0	7	0		
			MMC	20	24	63.2±7.2	11/9	12	0	8	0	0.2	2
Nilforushan (2011)	single	Iran	Ologen	7	13	59±12.6	4/3	7	0	0	0		
			MMC	7	14	59±12.6	4/3	7	0	0	0	0.2	3
Rosentreter (2010)	single	Germany	Ologen	10	12	62.8±9.5	8/12	na	na	0	na		
			MMC	10	12			na	na	0	na	0.2	3

M/F indicates male/female; MMC, mitomycin C; POAG, primary open-angle glaucoma; PACG, primary angle-closure glaucoma; PXFG, pseudoexfoliation glaucoma; con, concentration; min, minutes.

### 3. Quality Assessment

The agreement between the two reviewers’ quality assessment of the trials was scored by the κ coefficient (a measure of agreement), which was 0.85, with 92.1% observed agreement. The risk of bias in the RCTs is shown in Table 2. In four of the RCTs included in the systematic review, the investigators described a random component in the sequence generation process, such as referring to a random number table or using a computer random number generator or using random blocks. The remainder did not describe the specific methods of random sequence generation. Allocation concealment was either described or confirmed by author contact for two studies. Blinding of the personnel and observer was clearly described only by Cillino. Adequate assessment of each outcome and selective outcome reporting avoided were all reported in the randomized controlled trials, but none calculated the sample size before the trials.

**Table 2:** Evaluation of the risks of bias of RCTs included in the meta-analysis.

Trial (year)	Sequence Generation	Allocation Concealment	Blinding			Adequate asseement of each outcome	Selective reporting avoided	No Other Bias
			Patient	Personnel	Assessor			
Senthil (2013)	yes	no	no	no	no	yes	yes	yes
Marey (2013)	unclear	no	no	no	no	yes	yes	yes
Mitra (2012)	unclear	unclear	unclear	unclear	unclear	yes	yes	yes
Maheshwari (2012)	unclear	unclear	unclear	unclear	unclear	yes	yes	yes
Cillino (2011)	yes	yes	no	yes	yes	yes	yes	yes
Nilforushan (2011)	yes	no	no	no	no	yes	yes	yes
Rosentreter (2010)	yes	yes	no	no	no	yes	yes	yes

### 4. Reduction in the IOP (IOPR)

**Figure 2:**
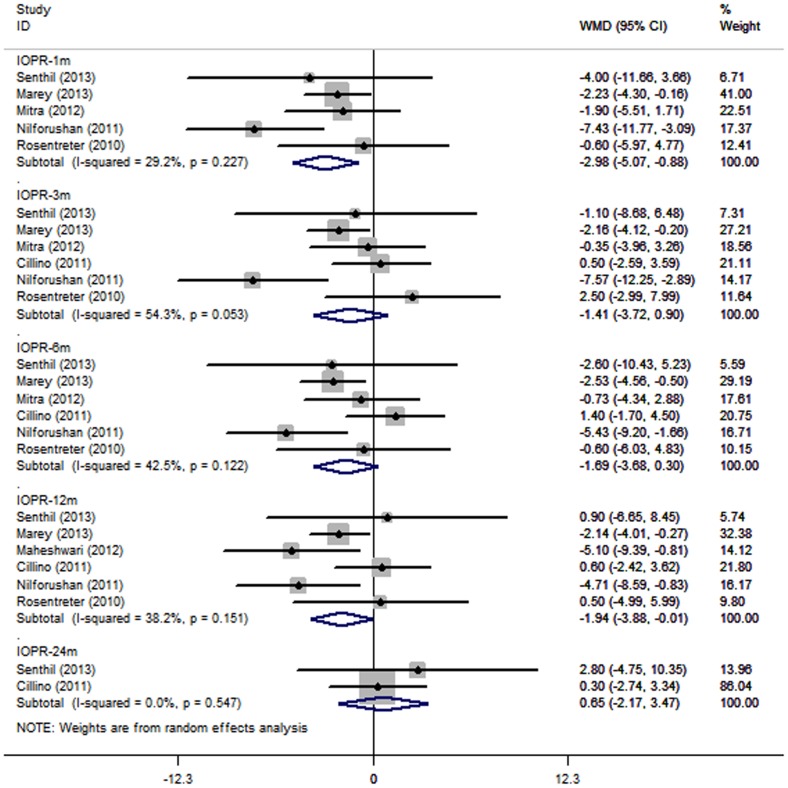
The weighted mean differences of the reduction in intraocular pressure between trabeculectomy with Ologen implant and with intraoperative mitomycin C. WMD indicates weighted mean difference, which were computed by using a random effects model.

Seven studies reported the IOPR at various time points, five of them at one month, six at three months, six at six months, six at 12 months, and two at 24 months. The IOP reduction was numerically smaller for the Ologen group at all intervals with the exception of 24 months. When comparing the Ologen group with the MMC group, the WMDs of the IOPR were −2.98 (95% Cl: −5.07 to −0.89) at one month, −1.41 (−3.72 to 0.91) at three months, −1.69 (−3.68 to 0.30) at six months, −1.94 (−3.88 to 0.01) at 12 months, and 0.65 (−2.17 to 0.47) at 24 months. There was no significant heterogeneity in these analyses, and the differences in IOPR were all not statistically significant, with the exception of one month and 12 months (Table 3 and Figure 2).

**Table 3:** The Reduction in Intraocular Pressure and Glaucoma Medication from Baseline Comparing Ologen implant With Intraoperative MMC in Patients Undergone Trabeculectomy.

Time	NO. of studies	WMD (95% CI)			Test for Heterogeneity			Test for Overall Effect	
		Estimate	ll	ul	?^2^	I^2^	P	Z	P
Reduction In Intraocular Pressure									
1m	5	−2.98	−5.07	−0.89	5.65	29.2%	0.227	2.79	0.005
3m	6	−1.41	−3.72	0.91	10.94	54.3%	0.053	1.19	0.233
6m	6	−1.69	−3.68	0.30	8.69	42.5%	0.122	1.67	0.096
12m	6	−1.94	−3.88	−0.01	8.10	38.2%	0.151	1.97	0.049
24m	2	0.65	−2.17	3.47	0.36	0.00%	0.547	0.45	0.652
Reduction in Glaucoma Medication									
6m	3	−0.31	−0.58	−0.045	0.08	0.00%	0.961	2.28	0.022
12m	2	−0.59	−1.36	0.19	0.00	0.00%	0.980	1.49	0.136
24m	2	0.01	−0.13	0.15	0.13	0.00%	0.723	0.10	0.923

Weighted mean differences were computed by using a random effects model. 95% CI indicates 95% confidence interval; MMC, mitomycin C.

### 5. Reduction in the Number of Glaucoma Medications

There was no significant difference in glaucoma medication reduction between the two groups except at one month (Table 3). The WMDs of reductions in the number of glaucoma medications after surgery (95% CI) were −0.31 (−0.58 to −0.045) at one month; −0.59 (−1.36 to 0.19) at 12 months; and 0.01 (−0.13 to 0.15) at 24 months, respectively. There was no evidence of heterogeneity for these outcomes (all P values >0.50; I^2^ = 0%).

### 6. Success Rates

Six studies reported the proportions of patients achieving the target endpoint IOP without anti-glaucoma medication at various time points, three of them at six months, five at 12 months, and two at 24 months. Ologen was associated with similar complete success rates compared with MMC at all time points (Table. 4), with the pooled RR being 1.19 (0.56 to 2.55) at six months, 0.74 (0.53 to 1.02) at 12 months, and 1.09 (0.77 to 1.56) at 24 months.

Six studies reported the proportions of patients achieving the target endpoint IOP with or without medications, no significant differences between groups were also found at three time points (Table 4) [pooled RR: 0.98 (0.87 to 1.10) at six months, 0.80 (0.57 to 1.11) at 12 months, and 1.06 (0.84 to 1.33) at 24 months]. There was no statistical evidence of heterogeneity across these studies (all P values >0.10; I^2^<50%).

**Table 4:** The Success Rate Comparing Ologen Implant and Intraoperative Mitomycin C in Patients Undergone Trabeculectomy.

	NO. of studies	Success Rate, n/N (%)		RR (95% CI)			Test for Heterogeneity			Test for Overall Effect	
		Ologen	MMC	Estimate	ll	ul	?^2^	I^2^	P	Z	P
Completed success rate											
6m	3	42/54	41/63	1.19	0.56	2.55	11.72	82.90%	0.003	0.45	0.652
12m	5	56/87	74/87	0.74	0.53	1.02	9.81	59.20%	0.044	1.84	0.066
24m	2	21/39	19/40	1.09	0.77	1.56	0.09	0.00%	0.765	0.49	0.623
Qualified success rate											
6m	3	47/54	57/63	0.98	0.87	1.10	1.52	0.00%	0.468	0.37	0.709
12m	3	31/46	39/47	0.80	0.57	1.11	3.60	44.40%	0.166	1.35	0.178
24m	2	24/39	23/40	1.06	0.84	1.33	0.00	0.00%	0.991	0.49	0.625

RR indicates relative risk, which were computed by using a random effects model. 95% CI indicates 95% confidence interval; n, number of patients achieving target endpoint intraocular pressure; N, number of patients; Ologen, trabeculectomy with Ologen implant; MMC, trabeculectomy with intraoperative mitomycin C.

### 7. Adverse Events

No significant differences in comparing the Ologen group and the MMC group were found in the incidence of bleb leak, hyphema, a shallow anterior chamber, hypotony, choroidal effusion, encapsulated bleb, blebitis, hypotony maculopathy, and implant exposure, with the pooled RRs being 1.08 (0.41 to 2.82), 1.78 (0.54 to 5.91), 0.85 (0.33 to 2.16), 1.02 (0.55 to 1.91), 0.74 (0.28 to 1.93), 1.68 (0.30 to 9.43), 1.13 (0.10 to 12.34), 0.50 (0.18 to 1.40), and 3.83 (0.16 to 90.53), respectively (Table 5).

**Table 5:** Adverse Events Comparing Ologen Group With MMC Group.

Adverse event	NO. of studies	Crude Rate, n/N (%)		RR (95% CI)			Test for Heterogeneity			Test for Overall Effect	
		Ologen	MMC	Estimate	ll	ul	?^2^	I^2^	P	Z	P
Bleb leak	5	7/107	7/116	1.08	0.41	2.82	3.17	0.00%	0.530	0.15	0.881
Hyphema	4	13/79	6/80	1.78	0.54	5.91	3.86	22.20%	0.277	0.95	0.344
Shallow anterior chamber	4	7/87	9/98	0.85	0.33	2.16	1.24	0.00%	0.743	0.34	0.732
Hypotony	3	10/57	10/66	1.02	0.55	1.91	0.03	0.00%	0.984	0.07	0.944
Choroidal effusion	3	6/49	9/50	0.74	0.28	1.93	1.74	0.00%	0.419	0.62	0.535
Encapsulated Bleb	2	3/38	2/46	1.68	0.30	9.43	0.06	0.00%	0.806	0.58	0.559
Blebitis	2	1/58	1/66	1.13	0.10	12.34	1.14	12.60%	0.285	0.10	0.921
Hypotony maculopathy	1	4/20	8/20	0.50	0.18	1.40	–	–	–	1.32	0.186
Implant Exposure	1	1/28	0/36	3.83	0.16	90.53	–	–	–	0.83	0.406

RR indicates relative risk, which were computed by using a random effects model. 95% CI indicates 95% confidence interval; n, number of patients achieving target endpoint intraocular pressure; N, number of patients; Ologen, trabeculectomy with Ologen implant; MMC, trabeculectomy with intraoperative mitomycin C.

### 8. Sensitivity Analysis and Publication Bias

To evaluate the robustness of the results, each study in the meta-analysis was excluded in turn to reflect the influence of individual studies on the pooled estimates of IOPR at three months, six months, and 12 months. The results indicated that the random-effect estimates before or after the deletion of any single study were generally similar, suggesting high stability in the meta-analysis results (data not shown). A funnel plot analysis indicated that the outcomes of IOPR at three months, six months, and 12 months were distributed symmetrically, showing no evidence of publication bias. Egger’s tests confirmed these results (Figure S1).

## Discussion

Glaucoma is the leading cause of irreversible blindness worldwide and represents a significant public health concern [30]. Surgical intervention is often needed in glaucoma patients who experience visual field deterioration or progressive optic neuropathy, despite maximum pharmacologic intervention, laser therapy, or both [1], [4]. Trabeculectomy remains the standard surgical procedure for the treatment of uncontrolled glaucoma worldwide. Its success rate and complications are well established. However, fibrosis of the sub-conjunctival tissue may lead to bleb failure, decreasing the long-term success of trabeculectomy. The introduction of adjunctive anti-metabolites such as MMC have improved the long-term success of trabeculectomy. However, MMC application is associated with higher long-term bleb-related complications [2], [3]. Aimed to improve the long-term surgical success of trabeculectomy but with fewer of the attendant complications of MMC, Ologen, a bioengineered porcine collagen, has been developed [2], [11], [31].

To the best of our knowledge, this is the first meta-analysis to explore the role of Ologen in trabeculectomy. The pooled results from the meta-analysis of seven RCTs using a random-effects model suggest similar postoperative behavior between the Ologen group and the MMC group, with stable IOP reduction and anti-glaucoma medications, indicating that the efficacy of the Ologen implant is analogous to that of MMC. The similarity between Ologen and MMC is further confirmed by their success rates at various times. In addition, the two agents contribute equally to adverse events. Sensitivity analysis suggested that the results were robust.

Ologen is composed of more than 90% lyophilized porcine collagen and <10% lyophilized glycos-aminoglycan with a pore size of 10–300 mm [32]. During trabeculectomy surgery, the implant is placed on top of the sclera flap before the conjunctiva is closed over it. After the implantation, the device completely degrades within 90–180 days [11]. The implant influences the aqueous flow by maintaining pressure on top of the sclera flap and by acting as a reservoir as the aqueous humour is absorbed into its porous structure. The collagen matrix attempts to provide a scaffold for the growth of fibroblasts and guides the fibroblasts to grow through the matrix pores in a random and diffuse fashion rather than in an organized way, thus altering tissue remodeling in the trabeculectomy wound and reducing scar formation [33]. This results in decreased scar formation and improved surgical success of trabeculectomy.

Ologen was numerically associated with somewhat lower IOP compared with MMC in the present study, but no significant differences were arrived. This may be related to differences inherent in each procedure. Ologen only functions as a wound modulator and does not have any antifibrotic properties to counter the scarring response. However,in previous human studies, Ologen implants used along with deep sclerectomy enhanced the success rates when compared to deep sclerectomy [13], [34]. Future randomized, controlled trials should help determine its place in glaucoma surgery.

Theoretically, the Ologen implant may protect against known MMC-associated complications. However, there was no significant difference in post-operative complications in the two groups, and no serious post-operative complications were noticed. In addition, there is a theoretical risk of increased inflammation in eyes with Ologen implants, as the implants are non-human (porcine) in origin [11]. However, no increase in inflammation was noticed, either in the form of increased anterior chamber reaction or hyperemic blebs. Further investigations are warranted to establish the long-term safety of the Ologen implant in glaucoma surgery.

The first strength of the present analysis is that we undertook meta-analyses by including only randomized clinical trials and excluding trials in which phacotrabeculectomy occurred. Furthermore, to avoid publication bias, we conducted not only an electronic search but also a manual search of the references of all the retrieved trials to identify all the potentially relevant articles, including published ones and non-published ones. The third strength is that only studies with a minimum follow-up period of six months were selected. Two independent co-authors judged the eligibility of articles and extracted data from the eligible articles, with discrepancies resolved after discussion by all of the authors. Only the series of the same patient group at the last endpoint were included in the present analysis.

This meta-analysis has several potential limitations that should be taken into account. One major limitation of this analysis was that patients were not stratified into high, medium, and low risk of trabeculectomy failure subgroups, which may produce more interesting results. Furthermore, although no significant heterogeneity was found, the studies were carried out with small or very small sample sizes, inadequate allocation concealment, or inadequate or no double blinding. These factors can greatly affect the interpretation of the results. The other limitations are the non-standardized assessment criteria of success. Success was defined as target endpoint IOP, and there were several different criteria for normal IOP, such as IOP≤18 mm Hg, ≤20 mm Hg, ≤21 mm Hg, and so on. Although such assessments of success are widely used as outcome measures in clinical trials, further research is still needed to determine fully their validity, reliability, and sensitivity to choose the best one. Finally, all participants in the studies were Caucasian, these results may not be generalized to other races such as Asians.

Nonetheless, the present study provides additional interesting clues that may be useful for future research on this important topic. First, future studies need to focus on other important clinical endpoints (e.g. visual acuity, visual field, and inflammation reactions) and biochemical indicators to understand the benefits, mechanisms, and role of Ologen in trabeculectomy better. In addition, only two RCTs with a modest sample size provided data on IOPR at 24 months; therefore, rigorous randomized controlled trials with long enough follow-up and large enough sample sizes are strongly recommended to evaluate further the real IOP-lowering effect of Ologen compared with MMC in trabeculectomy. Finally, further studies are needed to standardize a protocol (i.e. type of glaucoma, dosage and duration of MMC, and postoperative management) since variability exists in the literature.

## Conclusion

In summary, our systematic review indicates that trabeculectomy with Ologen is a safe and effective procedure in patients with glaucoma, but it does not seem to offer any significant advantages compared with trabeculectomy plus MMC. However, relevant evidence is still limited but is accumulating. Thus, studies with larger numbers of patients and longer follow-ups are urgently required to confirm these findings and to examine the safety and long-term outcomes of trabeculectomy with Ologen.

## Supporting Information

Figure S1 **Begg’s funnel plot for the IOPR of Ologen comparing to MMC in trabeculectomy.**
(DOC)

Table S1 **PRISMA checklist.**
(DOC)
