# Supplementary material for: Ologen Implant versus Mitomycin C for Trabeculectomy: A Systematic Review and Meta-Analysis
Source: PLoS One. 2014 Jan 20;9(1):e85782. doi: 10.1371/journal.pone.0085782 (PMC3896400; doi:10.1371/journal.pone.0085782)
Supplement: Figure S1 — Begg’s funnel plot for the IOPR of Ologen comparing to MMC in trabeculectomy. (DOC) [file pone.0085782.s001.doc]

**IOPR-3m**

(Begg's Test P=1.000; Egger's test P= 0.869)

**IOPR-6m**

(Begg's Test P=0.707; Egger's test P=0.901)

**IOPR-12m**

(Begg's Test P=1.000; Egger's test P=0.887)
